# Depressive Symptoms and Bone Mineral Density among Police Officers in a Northeastern US City

**DOI:** 10.5539/gjhs.v4n3p39

**Published:** 2012-05-01

**Authors:** Luenda E. Charles, Desta Fekedulegn, Diane B. Miller, Jean Wactawski-Wende, John M. Violanti, Michael E. Andrew, Cecil M Burchfiel

**Affiliations:** 1 Biostatistics and Epidemiology Branch, Health Effects Laboratory Division, National Institute for Occupational Safety and Health, Centers for Disease Control and Prevention, Morgantown, West Virginia, USA; 2 Toxicology and Molecular Biology Branch, Health Effects Laboratory Division, National Institute for Occupational Safety and Health, Centers for Disease Control and Prevention, Morgantown, West Virginia, USA; 3 Department of Social and Preventive Medicine, School of Public Health and Health Professions, University at Buffalo, The State University of New York. Buffalo, New York, USA

**Keywords:** depression, bone mineral density, osteoporosis, police officers, CES-D, gender

## Abstract

**Purpose:**

The purpose of this study was to examine the association between depressive symptoms and bone mineral density (BMD).

**Methods:**

Depressive symptoms were measured using the Center for Epidemiologic Studies Depression (CES-D) scale. BMD of total hip, femoral neck, anterio-posterior (AP) spine, wrist, and total body were measured by DXA using standardized procedures. Mean levels of BMD across gender-specific tertiles of CES-D score were obtained using ANOVA and ANCOVA.

**Results:**

Participants included 97 police officers (41 women; 29-64 years). Depressive symptoms were not associated with BMD at any site among men. However among women, mean BMD values decreased across increasing (worsening) tertiles of CES-D for the AP spine (low CES-D=1.22 ± 0.04; medium CES-D=1.05±0.04; high CES-D=1.03±0.04 g/cm^2^; p=0.035) and for the whole body (low=1.26±0.03; medium=1.20±0.03; high=1.11±0.03 g/cm^2^; p=0.018) after adjustment.

**Conclusions:**

Higher depressive symptoms were associated with lower BMD among female but not male officers.

## 1. Introduction

Osteoporosis, a disease characterized by low bone mineral density (BMD) and increased risk of fractures, is a major public health problem that is prevalent among the populations of developed and developing countries (Looker et al, 2010; Holt et al, 2002; Aggarwal et al, 2011; Lippurer, 2012; Kim et al, 2012). Among a sample of the U.S. population, there was a decline in the prevalence of low BMD in the femoral neck in 2005-2006 compared to 1988-1994, however, the absolute number of older adults with low BMD increased in 2005-2006 (Looker et al, 2010). Major risk factors for low BMD includeolder age, white race, female gender, cigarette smoking, use of medications such as corticosteroids, nutritional deficiencies (particularly vitamin D, calcium and magnesium), and low estrogen (Prentice, 2004; Bab & Yirmiya, 2010; Haney & Warden, 2008; Wynn et al, 2010; Falahati-Nini et al, 2000). Low BMD results in increased risk of fractures and increased medical costs, and has been associated with other factors including increased disability and premature death (Leboime et al, 2010; Broussard & Magnus, 2008).

Studies investigating the association between clinical depression and BMD have shown inverse relationships but these results have been inconsistent (Cizza et al, 2010; Wu et al, 2009). Police officers are a group that may be of particular interest to study as research has found that exposure to traumatic events and shift work is related to higher rates of depressive symptoms (Violanti and Aron, 1993; Wang et al, 2010). While an abundance of published studies on clinical depression and BMD exist, there are very few that studied depressive symptoms (Williams et al, 2011; Robbins et al, 2001; Tolea et al, 2007) and to our knowledge, none in police officers. Our objective was to examine the cross-sectional association between depressive symptoms and BMD among female and male police officers. We hypothesized that higher depressive symptoms will be associated with lower BMD among both women and men.

### 1.1 Background Information

Several biological mechanisms may account for associations between depression or depressive symptoms and BMD. A biological mechanism whereby depressive symptoms may be associated with BMD involves cortisol. Furlan and colleagues (2005) investigated whether cortisol mediated the relationship between bone density and depression in 19 postmenopausal women. They found that women who had been depressed at some time had significantly lower total lumbar and right femur DXA scores and higher salivary cortisol levels than women who had never been depressed. Salivary cortisol was inversely associated with the bone density Z scores at every measured bone site. Analyses to determine mediation suggested that 51-67% of the association between depression and bone density could be attributed to stress-induced changes in cortisol. The authors suggested that cortisol hyper-secretion in response to stress may partially explain the negative impact of depression on bone mineral density. These analyses were not conducted in the present study but they are worthwhile of future investigations.

Depressive symptoms may also be associated with BMD through increased systemic inflammation or oxidative stress. Results from an animal experiment showed that levels of the antioxidant superoxide dismutase in the plasma and femur were positively correlated with BMD of the femur thus indicating that oxidative stress was involved in bone loss (Zhang et al, 2011). In a case-control study, premenopausal women with major depressive disorder had increased levels of pro-inflammatory cytokines, decreased levels of anti-inflammatory cytokines, and a higher prevalence of low BMD at several sites compared to healthy control women (Eskandari et al, 2007). Many neuroendocrine hormones affect bone formation and/or bone resorption (Mezuk et al, 2008). Levels of many of the hormones that influence bone metabolism are altered in depression. Pro-inflammatory markers, tyrosine hydroxylase, and norepinephrine are other hormones that are elevated in depression and levels of these hormones are associated with reduced BMD (Mezuk et al, 2008). Depression is associated with decreased levels of the gonadal hormones estrogen and testosterone which are key regulators of bone formation. Depression is also known to stimulate the action of hormones that potentiate bone formation (e.g., insulin) (Mezuk et al, 2008).

Depressive symptoms may be associated with BMD through poor health behaviors as presented in the review by Mezuk and colleagues (2008). Depression is associated with smoking which inhibits estrogen activity and calcium absorption by the intestinal tract thereby resulting in lower BMD. Depression is associated with increased alcohol use and chronic alcohol use is also associated with low BMD through inhibition of bone cell proliferation and function. Depression is also associated with fatigue and physical inactivity; higher levels of physical activity are associated with increased BMD (Mezuk et al, 2008).

Osteoporosis and low bone density in many populations but especially among women is likely to become an even larger public health problem as the life span increases (Seeman, 2003; Cooper et al, 1992). Bone density in women can decrease precipitously after menopause (Seeman, 2003). This is due to factors associated with bone remodeling that increase osteoclast activity (which break down bone) and decrease osteoblast activity (which rebuild bone) as we age (Seeman, 2003). Investigation of risk factors and protective factors for BMD is important for everyone but may be most important in women.

## 2. Methods

### 2.1 Study Sample and Design

In 2001-2003, a pilot study was conducted to examine the associations between occupational stress and health outcomes of police officers from the Buffalo, New York Police department. All officers in the department were stratified by gender and 100 officers were randomly selected using a computer-generated random number table. Women have been under-represented in the law enforcement profession. Therefore, in order to obtain an adequate number of female officers for our study, females were oversampled by selecting a larger proportion of them than men during the random sampling procedure. One hundred percent of the random sample voluntarily agreed to participate in the study. No specific inclusion criteria were used for the study, other than that the participant would be a currently employed and sworn officer in the Buffalo NY police force. Examinations were conducted and data were collected using standardized procedures at the University at Buffalo, Center for Health Research. Officers were informed of the purpose of the study and were asked to read and sign informed consent forms. The study was approved by the Health Sciences Institutional Review Board at the University of Buffalo. The study is cross-sectional in design and details have been described previously (Violanti et al, 2006).

### 2.2 Depressive Symptoms

Depressive symptoms were measured utilizing the Center for Epidemiologic Studies Depression (CES-D) scale. The CES-D is a short scale (20 items on a 4-point scale) designed to measure symptoms of depression (e.g., poor appetite, restless sleep, sadness) in the general population (Radloff, 1977). It was designed for research purposes only. The 4-point scale represents how frequently each symptom occurred during the past seven days as follows: 0 (rarely or none of the time, less than 1 day); 1 (some or little of the time, 1-2 days); 2 (occasionally or a moderate amount of time, 3-4 days); and 3 (most or all of the time, 5-7 days). Four items were worded in the positive direction and these items were reverse coded. The positively worded items were: 1) I felt that I was just as good as other people; 2) I felt hopeful about the future; 3) I was happy; and 4) I enjoyed life. The CES-D is scored by assigning a value of 0 to 3 and summing the scores to obtain an overall score (range = 0 - 60). The CES-D has high internal consistency, acceptable reliability (Cronbach alpha = 0.86), and excellent concurrent validity by self-report and clinical criteria (Radloff, 1977).

### 2.3 Bone Mineral Density

Trained and certified technicians from the Osteoporosis Research Center at the University at Buffalo performed all bone density examinations. BMD was measured by dual-energy x-ray absorptiometry (DXA Hologic QDR^®^ 4500A machine; Hologic, Inc., Waltham, MA, USA) for the whole body and several body sites including the hip (neck and total hip regions), antero-posterior (AP) spine and the wrist (1/3 forearm). Anterior-posterior (AP) spine measures included lumbar vertebrae 1 to 4. The femur regions included the total hip and femoral neck. The forearm (both radius and ulna) was scanned with specific areas reported including the distal 1/3 region, mid region, ultra distal region, and total forearm region. Assessment of total body skeletal density and body composition (distribution of fat and lean) was performed. Results of BMD for each site were reported as g/cm^2^. Routine quality control was ascertained during this study and the coefficients of variation for each region were excellent and ranged from 0.22% (wrist) to 0.66% (AP spine).

### 2.4 Demographic and Lifestyle Characteristics

Questionnaires were used to ascertain demographic and other characteristics including age, race, gender, educational attainment, physical activity, and police rank. Police rank included patrol officers, sergeants, lieutenants, captains, detectives, and ‘other’. Officers were categorized as current, former and never smokers, and alcohol intake was based on the number of drinks reported per week using categories of 0, <1, 1-6, and >6 drinks per week. Smoking and alcohol intake information were obtained from a baseline study conducted two years earlier (1999-2001); 84 of the officers in the present study had participated at the baseline. Officers were weighed and height was measured according to standard protocols. Body mass index (BMI) was calculated as weight (in kilograms) divided by height (in meters) squared.

The officers reported the duration (hours per week, hours per weekend) and intensity (moderate, hard, very hard) of three types of physical activity (occupational, household, and sports) that they had engaged in during the previous seven days. These data were used to create the total hours of physical activity.

The officers also reported all medications that they were currently taking. The medications included groups known to decrease (e.g., corticosteroids, serotonin reuptake inhibitors) and increase BMD (e.g., estrogen, progestin, testosterone, and nutritional supplements such as calcium and vitamin D), as well as many medications that had no effect on BMD. Three dichotomous medication variables were created and used as covariates in the multivariate models: 1) increases BMD (yes vs. no); 2) decreases BMD (yes vs. no); and 3) affects BMD in either direction (yes vs. no). These medication variables were included one at a time (with other covariates) in the models.

### 2.5 Statistical Analyses

Only officers with complete data on the independent variable (CES-D score) and the dependent variable (BMD) (n = 97; 41 women and 56 men) were included in these statistical analyses. Descriptive statistics were used to summarize the demographic and life style characteristics of the study sample. Bivariate analyses included comparing mean CES-D score and mean BMD values across levels of the various demographic and life style characteristics using analysis of variance (ANOVA). Statistical models for the primary relationship of interest (i.e., assessing associations between CES-D score and BMD) were examined separately for women and men. Mean BMD values were obtained across gender-specific tertiles of CES-D score and compared using ANOVA and analysis of covariance (ANCOVA). We investigated several models where each covariate was added one at a time to compare the effect of the added variable to the unadjusted model. Only three models are presented in the tables. Simple and multiple linear regression analyses were used to test the significance of the linear trend in BMD across CES-D score. Residuals from the fitted models were tested for normality and homogeneity of variance using formal and graphical procedures. Potential confounders were selected based on whether the variable in question was associated with both CES-D score and BMD in the present study. All statistical analyses were performed using PROC GLM of the SAS software version 9.2 (SAS, Cary, NC).

## 3. Results

Characteristics of the study sample are presented in Table 1. Their ages ranged from 29 to 64 years with the majority of study participants being white (76.3%), under 50 years of age (75.3%), male (57.7%), and completing at least 4 years of college (55.7%).The prevalence of current smoking was 25% in women and 10.4% in men. A higher percentage of women were classified as having a normal BMI (51.2%) compared to 12.5% among men. As expected, the mean BMD values on every site measured were significantly higher among men than among women.

**Table 1:** Demographic and life style characteristics by gender

Characteristics	Women (n=41)		Men (n=56)		Total (n=97)		P-value
		
					
Race							0.083
White	31	75.6	43	76.8	74	76.3	
Black	10	24.4	8	14.3	18	18.6	
Hispanic	0	0.0	5	8.9	5	5.2	
Age group (years)							0.014
< 40	9	22.0	19	33.9	28	28.9	
40-49	26	63.4	19	33.9	45	46.4	
≥ 50	6	14.6	18	32.1	24	24.7	
Education							0.979
≤High school/GED	7	17.1	9	16.1	16	16.5	
College <4 yrs	11	26.8	16	28.6	27	27.8	
College 4+ yrs	23	56.1	31	55.3	54	55.7	
Years of service							0.691
1-5	8	19.5	11	19.6	19	19.6	
6-10	6	14.6	6	10.7	12	12.4	
11-15	11	26.8	11	19.6	22	22.7	
> 15	16	39.1	28	50.0	44	45.4	
Smoking status^a^							0.090
Current	9	25.0	5	10.4	14	16.7	
Former	15	41.7	17	35.4	32	38.1	
Never	12	33.3	26	54.2	38	45.2	
Body mass index (kg/m^2^)							<0.001
Normal (18.5-24.9)	21	51.2	7	12.5	28	28.9	
Overweight (25-29.9)	9	22.0	27	48.2	36	37.1	
Obese (≥ 30)	11	26.8	22	39.3	33	34.0	
Hours of physical activity^b^							0.535
Low (0-2)	12	34.2	13	27.7	25	30.5	
Medium (3-6.5)	15	42.9	18	38.3	33	40.2	
High (7-55.5)	8	22.9	16	34.0	24	29.3	
Meds that affect BMD	7	17.1	10	17.9	17	17.5	0.920
Meds that increase BMD	6	14.6	3	5.4	9	9.3	0.120
Meds that decrease BMD	3	7.3	8	14.3	11	11.3	0.285
Age (years)	43.8	5.7	44.0	8.8	43.9	7.6	0.895
CES-D score	9.8	8.8	7.7	6.4	8.6	7.6	0.213
Physical activity (hours)	4.6	4.1	9.4	12.3	7.4	9.9	0.016
Alcohol intake (drinks/wk)	2.0	3.1	3.9	4.7	3.1	4.2	0.032
BMD of total hip (g/cm^2^)	0.98	0.10	1.13	0.14	1.07	0.15	<0.0001
BMD of neck of femur (g/cm^2^)	0.87	0.11	0.97	0.13	0.92	0.13	<0.001
BMD of AP spine (g/cm^2^)	1.08	0.14	1.11	0.12	1.10	0.13	0.177
BMD of wrist (g/cm^2^)	0.59	0.04	0.70	0.05	0.65	0.07	<0.0001
BMD of whole body (g/cm^2^)	1.18	0.09	1.27	0.10	1.23	0.11	<0.0001

BMD: Bone mineral density.

a Smoking status variables were assessed at a baseline study 2-3 years prior (1999-2001) and data are available only for those seen at the baseline study (n=84).

b Physical activity hours was missing for 15 participants and the categories were based on tertiles.

Medication use: medication potentially affecting bone mineral density. Results are n(%) for categorical variables and mean ± SD for continuous variables. P-values for continuous variables comparing women and men are from Student’s *t*-tests. P-values for categorical variables comparing women and men are from chi-square or Fisher’s exact tests.

The following results are not presented in the tables. Among men only, CES-D score was negatively associated with education (p = 0.021) and positively associated with BMI (p = 0.009). BMD was inversely and significantly associated with age for most of the anatomical sites measured among both men and women, with those <40 years having the highest mean BMD and those ≥50 years having the lowest value. BMI was positively and significantly associated with BMD in the total hip (among men, p = 0.010) and in the femoral neck (among women, 0.018) with obese participants having the highest density compared to normal or overweight persons. Smoking status was significantly associated with BMD of the femoral neck among men (current smokers = 0.85 ± 0.06 g/cm^2^; former smokers = 0.99 ± 0.14 g/cm^2^; and never smokers = 0.93 ± 0.10 g/cm^2^; p = 0.048), and with the AP spine among women (current smokers = 1.03 ± 0.13 g/cm^2^; former smokers = 1.06 ± 0.13 g/cm^2^; and never smokers = 1.16 ± 0.13 g/cm^2^; p = 0.043). Compared to white women, black women had higher mean BMD of the wrist (0.62 vs. 0.58 g/cm^2^; p = 0.009) and of the whole body (1.23 vs. 1.16 g/cm^2^; p = 0.040). Mean BMD of the other sites were not significantly different between black and white women, nor were mean BMD of any location between black and white men significantly different.

Among men, no significant correlations were observed between CES-D score and BMD at any of the sites (Table 2). However, among women CES-D score was negatively and significantly correlated with BMD at the total hip (r = -0.344, p = 0.030), AP spine (r = -0.317, p = 0.046), and whole body (r = -0.397, p = 0.011) after adjustment for age.

**Table 2:** Age-adjusted correlation coefficients between CES-D score and bone mineral density (g/cm^2^) at measured sites, stratified by gender

Sites	Women			Men		
	
	N	r	p-value	N	r	p-value
Total hip	41	-0.344	0.030	56	-0.039	0.779
Femoral neck	41	-0.140	0.389	56	-0.110	0.422
AP spine	41	-0.317	0.046	56	0.042	0.759
Wrist	41	-0.194	0.231	56	-0.007	0.959
Whole body	41	-0.397	0.011	56	-0.031	0.825

r = Pearson’s correlation coefficient. p-values were obtained from Pearson’s correlation. AP = Anteroposterior

The mean values of BMD at the five anatomical sites are presented across tertiles of CES-D scores for women (Table 3). Mean values of BMD at the total hip decreased with increasing tertiles of CES-D scores before (p = 0.026) and after adjustment for age (p = 0.030), however this significant association was somewhat attenuated after further adjustment for education, smoking status, BMI, alcohol intake, physical activity, and medication/supplement use (low CES-D = 1.07 ± 0.03; medium CES-D = 0.98 ± 0.04; high CES-D = 0.95 ± 0.03 g/cm^2^; p = 0.111). CES-D score was inversely and significantly associated with BMD of the AP spine (low CES-D = 1.22 ± 0.04; medium CES-D = 1.05 ± 0.05; high CES-D = 1.03 ± 0.04 g/cm^2^; p = 0.035) and the whole body after full risk factor adjustment (low CES-D = 1.26 ± 0.03; medium CES-D = 1.20 ± 0.03; high CES-D = 1.11 ± 0.03 g/cm^2^; p = 0.018). In contrast, CES-D score was not significantly associated with BMD at any of the sites among men (Table 4). Inclusion of race/ethnicity in the models did not change any of the associations so this variable was excluded from the final models.

**Table 3:** Mean values of bone mineral density (g/cm^2^) at measured sites across tertiles of CES-D score for women

	Tertiles of CES-D score			B coeff., R^2^	p-value

	Low (0-3) *(n=13)*	Medium (4-10) *(n=14)*	High (11-34) *(n=14)*		
**Total hip**					
Model 1	1.02 ± 0.12	0.99 ± 0.08	0.94 ± 0.09	-0.004, 0.121	0.026
Model 2	1.03 ± 0.03	0.99 ± 0.02	0.94 ± 0.02	-0.004, 0.203	0.030
Model 3	1.07 ± 0.03	0.98 ± 0.04	0.95 ± 0.03	-0.004, 0.122	0.111
**Femoral neck**					
Model 1	0.90 ± 0.13	0.86 ± 0.10	0.84 ± 0.10	-0.001, 0.025	0.321
Model 2	0.91 ± 0.03	0.85 ± 0.03	0.84 ± 0.03	-0.002, 0.108	0.389
Model 3	0.93 ± 0.04	0.85 ± 0.04	0.84 ± 0.03	-0.002, 0.143	0.401
**AP spine**					
Model 1	1.15 ± 0.15	1.05 ± 0.12	1.04 ± 0.12	-0.005, 0.105	0.039
Model 2	1.16 ± 0.04	1.04 ± 0.04	1.03 ± 0.04	-0.005, 0.069	0.046
Model 3	1.22 ± 0.04	1.05 ± 0.05	1.03 ± 0.04	-0.006, 0.133	0.035
**Wrist**					
Model 1	0.60 ± 0.04	0.59 ± 0.04	0.58 ± 0.04	-0.001, 0.042	0.198
Model 2	0.61 ± 0.01	0.59 ± 0.01	0.58 ± 0.01	-0.001, 0.032	0.231
Model 3	0.60 ± 0.02	0.60 ± 0.02	0.57 ± 0.01	-0.001, 0.133	0.215
**Whole body**					
Model 1	1.23 ± 0.10	1.17 ± 0.09	1.14 ± 0.09	-0.004, 0.163	0.009
Model 2	1.23 ± 0.02	1.16 ± 0.02	1.14 ± 0.02	-0.004, 0.163	0.011
Model 3	1.26 ± 0.03	1.20 ± 0.03	1.11 ± 0.03	-0.005, 0.147	0.018

Model 1: Unadjusted Model 2: Adjusted for age Model 3: Adjusted for age, education, smoking status, BMI, alcohol intake, physical activity, and use of medication that may affect bone density. P-values for trend were obtained from linear regression. Results are means ± standard deviations (SD) for model 1 and means ± standard errors (SE) for all other models. In model 3, information on smoking status was not available for 5 women.

**Table 4:** Mean values of bone mineral density (g/cm^2^) at measured sites across tertiles of CES-D score for men

	Tertiles of CES-D score			B coeff., R^2^	p-value

	Low (0-4) *(n=20)*	Medium (5-8) *(n=19)*	High (9-25) *(n=17)*		
**Total hip**					
Model 1	1.13 ± 0.13	1.15 ± 0.17	1.11 ± 0.12	-0.002, 0.005	0.614
Model 2	1.13 ± 0.03	1.15 ± 0.03	1.11 ± 0.03	-0.001, -0.001	0.779
Model 3	1.08 ± 0.04	1.16 ± 0.04	1.07 ± 0.04	-0.001, 0.038	0.879
**Femoral neck**					
Model 1	0.97 ± 0.12	0.99 ± 0.15	0.94 ± 0.12	-0.003, 0.024	0.255
Model 2	0.97 ± 0.03	0.99 ± 0.03	0.94 ± 0.03	-0.002, 0.065	0.422
Model 3	0.94 ± 0.04	0.95 ± 0.04	0.89 ± 0.04	-0.003, 0.275	0.330
**AP spine**					
Model 1	1.10 ± 0.11	1.12 ± 0.14	1.12 ± 0.11	0.001, 0.001	0.821
Model 2	1.10 ± 0.03	1.12 ± 0.03	1.13 ± 0.03	0.001, -0.032	0.759
Model 3	1.07 ± 0.05	1.14 ± 0.05	1.07 ± 0.05	-0.001, -0.117	0.899
**Wrist**					
Model 1	0.69 ± 0.05	0.70 ± 0.06	0.70 ± 0.05	-0.001, 0.001	0.798
Model 2	0.69 ± 0.01	0.70 ± 0.01	0.70 ± 0.01	-0.001, -0.010	0.959
Model 3	0.69 ± 0.02	0.70 ± 0.02	0.70 ± 0.02	-0.001, -0.204	0.636
**Whole body**					
Model 1	1.25 ± 0.09	1.28 ± 0.12	1.27 ± 0.09	-0.001, 0.002	0.758
Model 2	1.25 ± 0.02	1.28 ± 0.02	1.27 ± 0.03	-0.001, -0.032	0.825
Model 3	1.24 ± 0.04	1.30 ± 0.04	1.24 ± 0.04	-0.002, -0.125	0.577

Model 1: Unadjusted Model 2: Adjusted for age Model 3: Adjusted for age, education, smoking status, BMI, alcohol intake, physical activity, and use of medication that may affect bone density. P-values for trend were obtained from linear regression. Results are means ± standard deviations (SD) for model 1 and means ± standard errors (SE) for all other models. In model 3, information on smoking status was not available for 8 men.

Sensitivity analyses were conducted between depressive symptoms and BMD as follows: (a) by excluding all officers (7 women and 10 men) who reported taking any medications that affected BMD; (b) by including in the multivariate model adjustment for medications and supplements that increase BMD; and (c) by including in the multivariate model adjustment for medications and supplements that decrease BMD. When we excluded all officers who took medication or supplements known to affect BMD, the association among men was unchanged, however among women, CES-D score was inversely and significantly associated with BMD at all locations except for wrist. When medications and supplements that either increased or decreased BMD were included in the model, the results among women were similar to that obtained in Table 3. Regardless of the sensitivity analyses attempted, CES-D score was not associated with BMD at any location among men.

## 4. Discussion

In this cohort of female and male police officers, we examined the association between depressive symptoms and BMD at five anatomical sites. To our knowledge, this is the first investigation of its kind among an occupational group that is exposed to a wide variety of conventional and unconventional work place stressors.

Among women, higher levels of depressive symptoms were significantly associated with lower mean levels of BMD for the AP spine and the whole body, after full adjustment. Inverse associations were also observed between depressive symptoms and BMD of the hip although this association was not statistically significant. We did not observe any significant associations with BMD at any of the sites among men. Police work has been shown to be more stressful to female officers because they may experience sexual harassment and racial discrimination which most white male officers do not usually experience (Hassell & Brandl, 2009). Females may have more non-occupational responsibilities such as childcare and household chores. Female officers may therefore be more vulnerable to higher levels of depression and consequently experience a higher prevalence of decreased BMD (Altindag et al, 2007).

Our results of inverse associations between depressive symptoms and BMD are supported by other work. Clinical depression and depressive symptoms have been shown in previous studies to be associated with lower BMD, and the association sometimes differed by gender and among the various anatomical sites (Cizza et al, 2010; Wu et al, 2009; Williams et al, 2011; Altindag et al, 2007; Mussolino et al, 2004; Eskandari et al, 2007). Other studies did not find an association between depression or depressive symptoms and lower BMD (Yazici et al, 2005; Whooley et al, 2004; Reginster et al, 1999). The differences among the studies may be due to several factors. Several of the studies assessed depressive symptoms using questionnaires other than the CES-D (e.g., Hamilton Depression Scale, Montgomery Asberg Depression Rating Scale, General Health Questionnaire, Geriatric Depression Scale, General Well-Being Schedule, etc.). Study designs, use of patients versus community populations, control of different confounding variables, and sample sizes (ranging from <100 to >6000) also varied in the studies and may have contributed to differences in the findings. Police officers in this study, because of their profession, may be particularly exposed to events leading to depression.

When medications and supplements that either increase or decrease BMD were adjusted for as potential confounders in the models, the results among women were similar to that reported above. However, when we analyzed the association between CES-D score and BMD after excluding the 17 officers who took medications or supplements known to affect BMD in any direction, the associations with BMD were stronger among women at all sites measured except for the wrist. Additional investigation into this difference showed that the results from the model that excluded these 17 officers were overly influenced by a single officer, therefore, results presented included these 17 officers. Regardless of the type of analyses employed, CES-D score was not associated with BMD at any location among men. The absence of an association among men may be due to the fact that osteoporosis develops in a more gradual manner in men than in women (Seeman, 2003). Bone loss occurs due to a decrease in the volume of bone formed rather than an increase in the volume of bone removed during remodeling. In menopausal women, bone loss increases rapidly because decreased estrogen production increases the rate of bone remodeling. However, this rapid bone loss is not present in men because the trabecular surface that is available for remodeling is better preserved (Seeman, 2003).

The cross-sectional design of the study was a limitation in that causal inferences could not be drawn. Some police officers may have underestimated their alcohol consumption due to the stigma attached to this habit. If this were the case, information bias may have occurred but the influence of this covariate in the multivariate model is likely to be minimal. There was no information on hormonal levels so we were unable to adjust for this potentially confounding variable. In addition, we were unable to assess effect modification for variables other than gender due to a relatively small sample size. Although characteristics of officers may vary across police departments, the findings of this study may be generalized to other urban departments of a similar size. However, there are several strengths to this study. Although use of a cross-sectional design prevents us from making causal inferences, the cross-sectional study design allows epidemiological investigations to be conducted quickly and relatively inexpensively. To our knowledge, this is the first study to investigate the association between depressive symptoms and BMD in female and male police officers. We had standardized assessment of CES-D score (Radloff, 1977) and objective assessment of BMD. In addition, BMD data were available for five anatomical sites. We were able to adjust for medications and supplements that affect BMD. Studies have provided evidence that production of excess glucocorticoid or ingestion of corticosteroids and several other drugs decrease BMD and increase bone resorption (Chiodini et al, 1998; Pearce et al, 1998; Lovas et al, 2009; Man and Sin, 2009; Schubert, 2009), and that a healthy diet with increased supplementation of several vitamins and minerals are necessary for bone health (Prentice, 2004). Finally, given that policing has historically been a male-dominated profession, assessment of women, due to over-sampling, allowed for a unique examination of women officers in our study. The topic is highly relevant for all women given the higher burden and related consequences of osteoporosis among this group.

In conclusion, our findings show that depressive symptoms, as measured by CES-D, are associated with lower levels of BMD among women but not among men. Researchers estimate that the annual number of fractures in the world will reach 2.6 million by 2025 and 4.5 million by 2050 in comparison with 1.7 million cases in 1990 (Gulberg et al, 1997). According to the National Institutes of Health (NIH, 2000), osteoporosis is largely preventable. One must take regular steps to optimize bone health throughout life to prevent osteoporosis and its devastating consequences. Protective measures include dietary modifications (e.g., increased intake of calcium, vitamin D, etc.), implementation of stress management programs, low to moderate alcohol consumption, frequent involvement in bone strengthening exercises, and judicious use of corticosteroid therapy and other medications that adversely affect bone density. Future studies should investigate the association between depressive symptoms and BMD, especially in groups with high proportions of depressive symptoms, using a longitudinal study design and larger samples. Evaluation and implementation of effective interventions are also warranted.

**Abbreviations**
ANOVA: Analysis of variance
ANCOVA: Analysis of covariance
AP spine: Anterior-posterior spine
BMD: Bone mineral density
BMI: Body mass index
CES-D: Center for Epidemiologic Studies Depression scale
DXA: Dual Energy X-ray Absorptiometry
